# Is the residual approach reliable to estimate resilience to Alzheimer’s disease pathology? Comparison among different methods

**DOI:** 10.1002/alz.092275

**Published:** 2025-01-09

**Authors:** Sophie Mutel, Lara Quattrocchi, Daniele Altomare, Max Scheffler, Christian Chicherio, Kaj Blennow, Henrik Zetterberg, Valentina Garibotto, Nicholas J. Ashton, Giovanni B Frisoni, Federica Ribaldi

**Affiliations:** ^1^ Geneva Memory Center, Department of Rehabilitation and Geriatrics, Geneva University Hospitals, Geneva, Genève Switzerland; ^2^ University of Geneva, Geneva Switzerland; ^3^ University of Brescia, Brescia Italy; ^4^ Division of Radiology, Geneva University Hospitals, Geneva Switzerland; ^5^ Geneva University Hospitals, Geneva Switzerland; ^6^ Institute of Neuroscience and Physiology, Department of Psychiatry and Neurochemistry, The Sahlgrenska Academy at University of Gothenburg, Mölndal, ‐ Sweden; ^7^ Department of Psychiatry and Neurochemistry, Institute of Neuroscience and Physiology, The Sahlgrenska Academy, University of Gothenburg, Mölndal, Gothenburg Sweden; ^8^ Division of Nuclear Medicine and Molecular Imaging, Geneva University Hospitals, Geneva Switzerland; ^9^ Laboratory of Neuroimaging of Aging (LANVIE), University of Geneva, Geneva Switzerland

## Abstract

**Background:**

Resilience, the ability to maintain normal cognition (cognitive resilience, CR) or brain integrity (brain resilience, BR) despite neuropathological burden, is often quantified using the residual approach. This method calculates residuals from a linear regression where the dependent variable is brain volume or cognition, and the independent variable is neuropathology. Residuals can be corrected to render them independent from the dependent variable, but comparative analyses of residual correction methods within memory clinic populations are scarce. Our study aims to bridge this gap by comparing non‐corrected and corrected residual approaches.

**Methods:**

112 MCI patients from the Geneva Memory Center who underwent amyloid‐PET (A), tau‐PET (T), MRI (N), and clinical and neuropsychological assessments were included. Standardized residuals were extracted from linear regression models between hippocampal volume and AT (BR) or MMSE scores and ATN (CR). We compared: (i) non‐corrected residuals (nBR and nCR); (ii) residuals corrected by regressing the dependent variable out of the residuals (cBR and cCR); (iii) non‐corrected residuals with the dependent variable included as covariate (covBR and covCR). Associations between CR or BR and demographic, clinical, cognitive, and biomarker variables were investigated. Linear mixed models explored the impact of CR and BR on cognitive trajectories over time. Results were compared with non‐residual based statistical models.

**Results:**

Corrected and non‐corrected approaches yielded significantly different results. Several factors were associated with nBR, but not with cBR or covBR, such as age (nBR: β = ‐0.399, p < 0.001; cBR: β = ‐0.048, p = 0.641; covBR: β = ‐0.012, p = 0.614). Conversely, several factors were associated with cBR and covBR, bur not nBR, such as female sex (nBR: β = ‐0.102, p = 0.619; cBR: β = ‐0.506, p = 0.012; covBR: β = ‐0.108, p = 0.013). Similar results were observed with CR. Longitudinal assessments also indicated differences in the relationship between corrected/non‐corrected resilience and cognitive trajectories. For example, there was a significant interaction of time x cBR on MMSE scores (p < 0.001), but not of time x nBR (p = 0.226)

**Conclusion:**

Researchers should be aware that the choice of residual correction method significantly influences study conclusions. Our findings emphasize the necessity of adopting sophisticated modeling approaches to accurately capture resilience.

